# Supplementary material for: Antigen concentration, viral load, and test performance for SARS-CoV-2 in multiple specimen types
Source: PLoS One. 2023 Jul 19;18(7):e0287814. doi: 10.1371/journal.pone.0287814 (PMC10355390; doi:10.1371/journal.pone.0287814)
Supplement: S1 Table — Descriptions are of participant symptoms, regardless of test positivity. (DOCX) [file pone.0287814.s001.docx]

**Supplementary Material**

**Table S1. Case definitions for the study.** Descriptions are of participant symptoms, regardless of test positivity.

| **Category** | **Description** |
| --- | --- |
| Symptomatic | Participants presenting with cough, shortness of breath, difficulty breathing, or at least two of the following symptoms at the time of sampling: fever, chills, rigor, myalgia, headache, sore throat, new olfactory or taste disorder |
| Oligosymptomatic | Participants who presented with one or more mild symptoms but did not fit the symptomatic case definition and reported no care seeking or changes to behavior |
| Asymptomatic | Participants with no symptoms at the time of sampling |
