# Supplementary material for: Antigen concentration, viral load, and test performance for SARS-CoV-2 in multiple specimen types
Source: PLoS One. 2023 Jul 19;18(7):e0287814. doi: 10.1371/journal.pone.0287814 (PMC10355390; doi:10.1371/journal.pone.0287814)
Supplement: S2 Table — Result totals are shown for the study participants for index cases, household contacts, and non-household contacts. The proportion of samples selected and run for antigen testing from each participant is shown for nasopharyngeal swab (NPS), anterior nares swab (ANS), and saliva. The lysis buffer for the STANDARD Q ANS specimen was used for antigen concentration determination in the ANS specimen. Because the 64 household contacts had multiple timepoints per individual, test results are reported for the combined individual and timepoint together for a total of 224 samplings). Positive and negative classifications correspond to available test results from laboratory PCR results, SalivaDirect, STANDARD Q Saliva, LumiraDx, and STANDARD Q point-of-care (anterior nasal) and exclude antigen concentration measurement results. Totals are listed with breakdowns from those totals of number of Sympomatic (S), Oligosymptomatic (O), Asymptomatic (A). (DOCX) [file pone.0287814.s002.docx]

**Table S2. Summary of SARS-CoV-2 cases, associated specimens, and number of antigen results.** Result totals are shown for the study participants for index cases, household contacts, and non-household contacts. The proportion of samples selected and run for antigen testing from each participant is shown for nasopharyngeal swab (NPS), anterior nares swab (ANS), and saliva. The lysis buffer for the STANDARD Q ANS specimen was used for antigen concentration determination in the ANS specimen. Because the 64 household contacts had multiple timepoints per individual, test results are reported for the combined individual and timepoint together for a total of 224 samplings). Positive and negative classifications correspond to available test results from laboratory PCR results, SalivaDirect, STANDARD Q Saliva, LumiraDx, and STANDARD Q point-of-care (anterior nasal) and exclude antigen concentration measurement results. Totals are listed with breakdowns from those totals of number of Symptomatic (S), Oligosymptomatic (O), Asymptomatic (A).

| **Participant type** | **Negative by all tests** | **Positive by only one test** | **Positive by only two tests** | **Positive by three or more tests** | **Sample type** | **Selected for antigen concentration testing** | **Insufficient or missing sample** | **Antigen concentration determined** |
| --- | --- | --- | --- | --- | --- | --- | --- | --- |
| **Index cases**  **N=50** | 0 | 0 | 0 | 50  S:50  O:0  A:0 | **NPS** | 50 | 0 | 50 |
|  |  |  |  |  | **ANS** | 50 | 2 | 48 |
|  |  |  |  |  | **Saliva** | 50 | 0 | 50 |
| **Close Contacts,**  **Household**  **N= 64 individual**  **224 individuals and timepoint combinations** | 143  S: 6  O: 8  A: 129 | 19  S: 1  O: 1  A: 17 | 15  S: 5  O: 1  A: 9 | 47  S: 33  O: 4  A: 10 | **NPS** | 191 | 28  (no NPS taken) | 163 |
|  |  |  |  |  | **ANS** | 191 | 5 | 186 |
|  |  |  |  |  | **Saliva** | 191 | 8 | 183 |
| **Close Contacts, Non-household**  **N= 150** | 117  S: 19  O: 11  A: 87 | 6  S: 0  O: 1  A: 5 | 7  S: 1  O: 2  A: 4 | 20  S: 15  O: 4  A: 1 | **NPS** | 94 | 0 | 94 |
|  |  |  |  |  | **ANS** | 94 | 1 | 93 |
|  |  |  |  |  | **Saliva** | 94 | 2 | 92  + 2 not initially selected = 94 |
