# Supplementary material for: Antigen concentration, viral load, and test performance for SARS-CoV-2 in multiple specimen types
Source: PLoS One. 2023 Jul 19;18(7):e0287814. doi: 10.1371/journal.pone.0287814 (PMC10355390; doi:10.1371/journal.pone.0287814)
Supplement: S3 Table — For binary test results, 1 = positive, 0 = negative. Test line intensity for STANDARD Q Nasal and Saliva tests were reported according to 0–4 scale shown in S1 Fig. Lumira Signal refers to signal output from the LumiraDx instrument. (DOCX) [file pone.0287814.s003.docx]

**Table S3**. **Discrepancy analysis of specimens with a high NPS viral load but a negative test result from the antigen test conducted on the ANS specimen**. For binary test results, 1 = positive, 0 = negative. Test line intensity for STANDARD Q Nasal and Saliva tests were reported according to 0-4 scale shown in Figure S1. Lumira Signal refers to signal output from the LumiraDx instrument.

| Participant ID | Visit # | PCR N gene, Ct | Viral genome copies/mL | Sequencing clade | | N-antigen sense mutations | STANDARD Q Saliva Result | STANDARD Q Saliva Intensity | Lumira Result | Lumira Signal | STANDARD Q Nasal Result | STANDARD Q Nasal Intensity | Antigen Concentration, pg/mL in Standard Q buffer | |
| --- | --- | --- | --- | --- | --- | --- | --- | --- | --- | --- | --- | --- | --- | --- |
| CC-088 | 1 | 22.2 | 30188058 | Gamma, V3 | | P80R, R203K, G204R | 1 | 4 | 1 | 1181.6 | 0 | 0 | 29.2 | |
| CC-209 | 1 | 23.5 | 2627412 | not available | | not available | 0 | 0 | 0 | 252.4 | 0 | 0 | 0.0 | |
| CC-135 | 1 | 23.7 | 10720679 | Gamma, V3 | | P80R, R203K, G204R, T391I | 1 | 1 | 0 | 518.5 | 1 | 1 | 20.6 | |
| CC-017 | 3 | 23.8 | 10225694 | Gamma, V3 | | P80R, R203K, G204R, Q240L | 0 | 0 | 0 | 245.9 | 0 | 0 | 0.0 | |
| CC-163 | 1 | 25.4 | 3267859 | Gamma, V3 | | P80R, R203K, G204R, P344L | 0 | 0 | 0 | 555.1 | 1 | 1 | 4.2 | |
| CC-114 | 1 | 25.9 | 2319762 | Gamma, V3 | | P80R, R203K, G204Q | 0 | 0 | 0 | 321.6 | 1 | 1 | 258.9 | |
| CC-043 | 1 | 25.7 | 2593367 | Gamma, V3 | | P80R, R203K, G204R | 1 | 3 | 0 | 293.5 | 0 | 0 | 0.0 | |
| CC-054 | 1 | 25.9 | 2341293 | Gamma, V3 | | P80R, T135I, R203K, G204R | 1 | 1 | 0 | 780.1 | 1 | 1 | 84.8 | |
| IND-037 | 1 | 27.1 | 1017678 | Gamma, V3 | P80R, R203K, G204R, S413I | | 0 | 0 | 0 | 661.4 | 1 | 1 | 39.9 |  |
| CC-009 | 2 | 29.5 | 717496 | not available | not available | | 0 | 0 | 1 | 1690.2 | 0 | 0 | 79.1 |  |
| CC-200 | 1 | 29.6 | 158489 | not available | not available | | 0 | 0 | 0 | 255.9 | 0 | 0 | 0.0 |  |
| CC-029 | 2 | 34.3 | 16670 | not available | not available | | 1 | 2 | 0 | 243.4 | 0 | 0 | 0.0 |  |
| CC-082 | 1 | 33.6 | 10755 | not available | not available | | 0 | 0 | 0 | 185.7 | 0 | 0 | 7.6 |  |
| CC-149 | 1 | 33.5 | 11497 | not available | not available | | 0 | 0 | 0 | 232.2 | 0 | 0 | 0.0 |  |
