# Supplementary material for: Antigen concentration, viral load, and test performance for SARS-CoV-2 in multiple specimen types
Source: PLoS One. 2023 Jul 19;18(7):e0287814. doi: 10.1371/journal.pone.0287814 (PMC10355390; doi:10.1371/journal.pone.0287814)

**Figure S1. Score card for line intensity on the STANDARD Q COVID-19 Ag test (0 refers to no visible test line, or negative score) for nasal and saliva.**


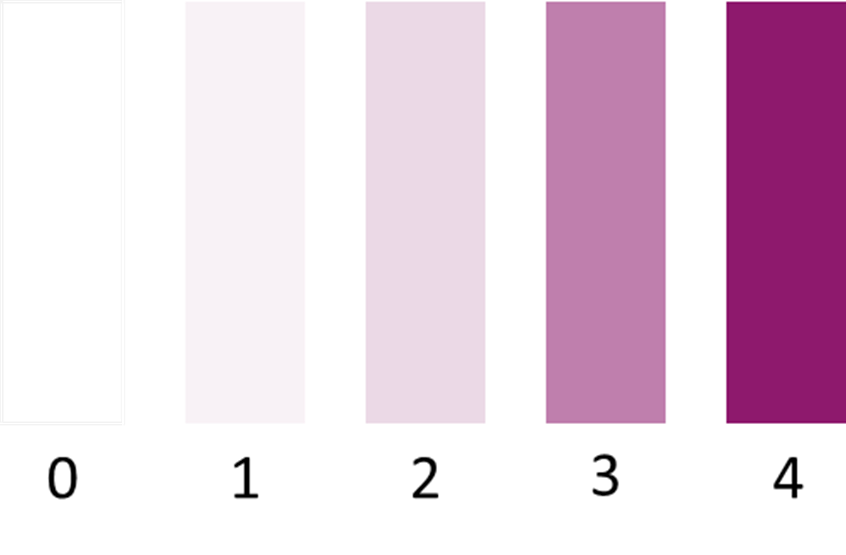

Supplement: S1 Fig — (DOCX) [file pone.0287814.s004.docx]
